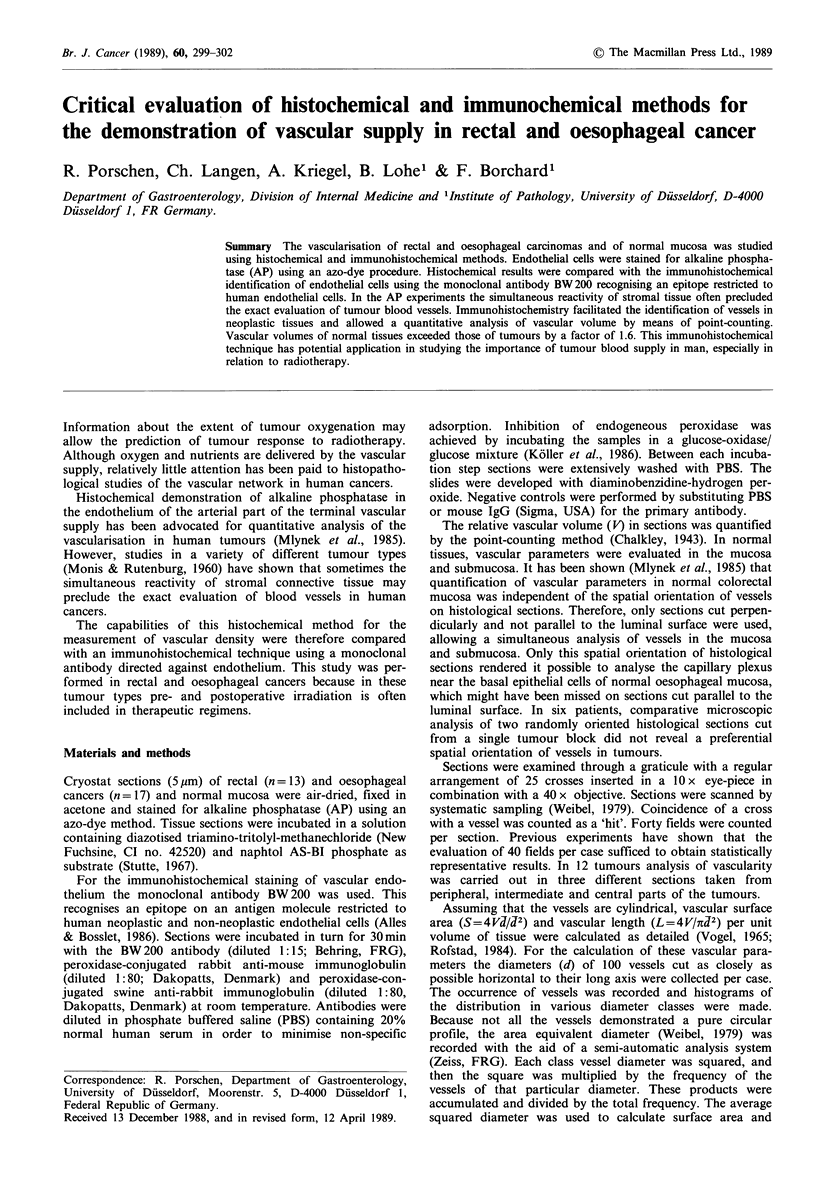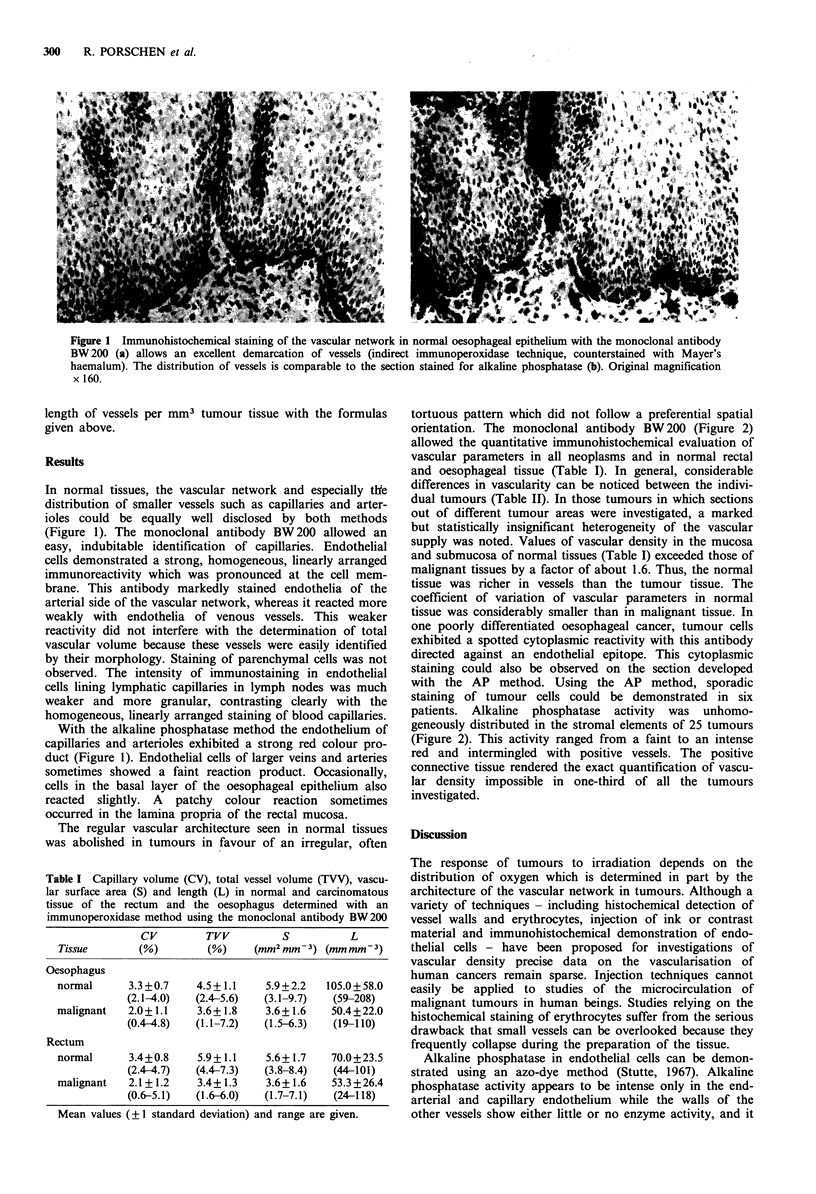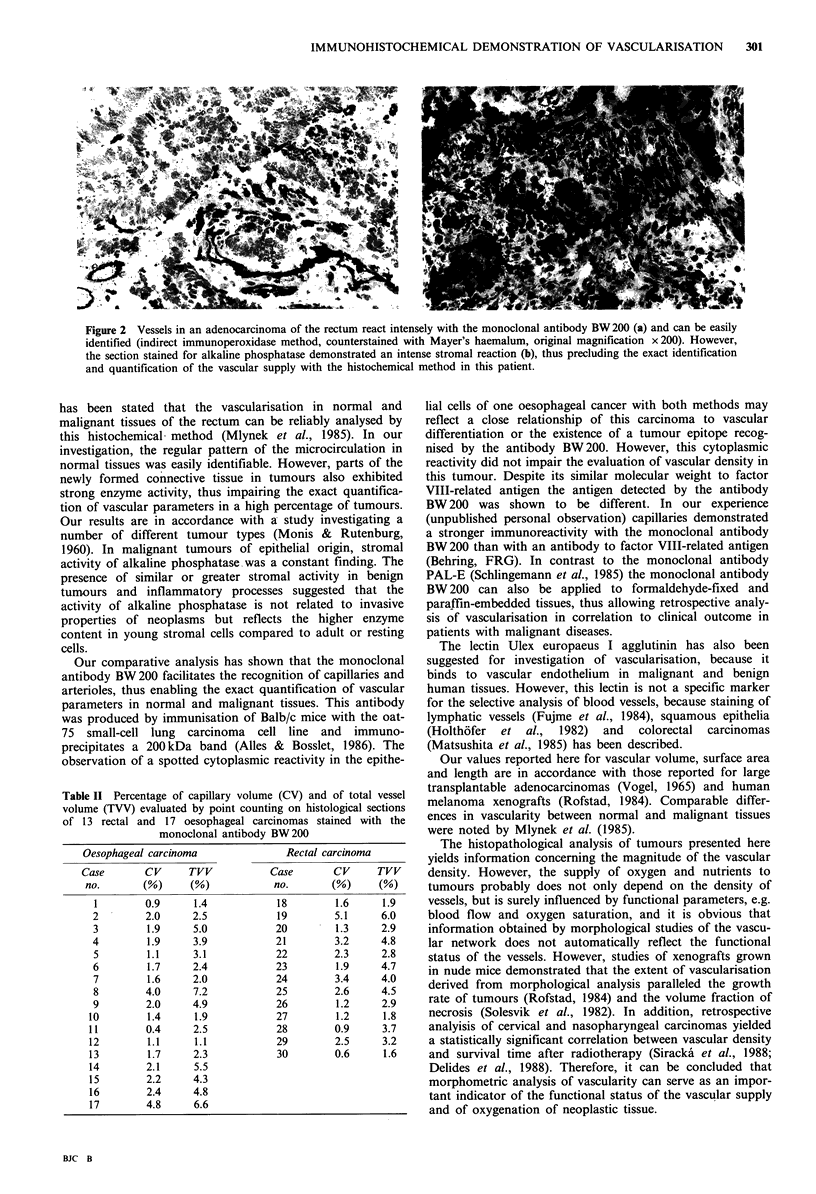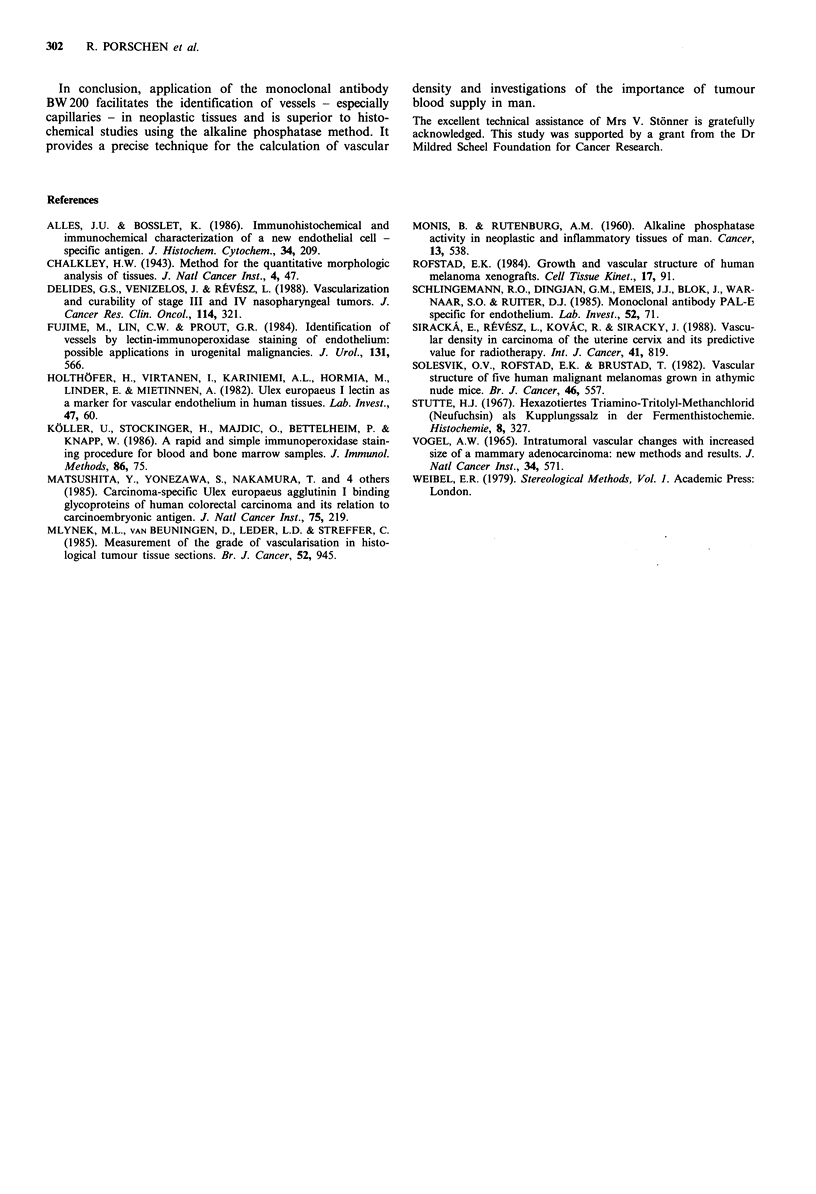# Critical evaluation of histochemical and immunochemical methods for the demonstration of vascular supply in rectal and oesophageal cancer.

**DOI:** 10.1038/bjc.1989.274

**Published:** 1989-09

**Authors:** R. Porschen, C. Langen, A. Kriegel, B. Lohe, F. Borchard

**Affiliations:** Department of Gastroenterology, University of DÃ¼sseldorf, FR Germany.

## Abstract

**Images:**


*Br. J. Cancer* (1989), **60**, 299–302
© The Macmillan Press Ltd., 1989

### Critical evaluation of histochemical and immunochemical methods for the demonstration of vascular supply in rectal and oesophageal cancer

R. Porschen, Ch. Langen, A. Kriegel, B. Lohe[1] & F. Borchard[1]

*Department of Gastroenterology, Division of Internal Medicine and [1]Institute of Pathology, University of Düsseldorf, D-4000 Düsseldorf 1, FR Germany.*

**Summary** The vascularisation of rectal and oesophageal carcinomas and of normal mucosa was studied using histochemical and immunohistochemical methods. Endothelial cells were stained for alkaline phosphatase (AP) using an azo-dye procedure. Histochemical results were compared with the immunohistochemical identification of endothelial cells using the monoclonal antibody BW200 recognising an epitope restricted to human endothelial cells. In the AP experiments the simultaneous reactivity of stromal tissue often precluded the exact evaluation of tumour blood vessels. Immunohistochemistry facilitated the identification of vessels in neoplastic tissues and allowed a quantitative analysis of vascular volume by means of point-counting. Vascular volumes of normal tissues exceeded those of tumours by a factor of 1.6. This immunohistochemical technique has potential application in studying the importance of tumour blood supply in man, especially in relation to radiotherapy.

Information about the extent of tumour oxygenation may allow the prediction of tumour response to radiotherapy. Although oxygen and nutrients are delivered by the vascular supply, relatively little attention has been paid to histopathological studies of the vascular network in human cancers.

Histochemical demonstration of alkaline phosphatase in the endothelium of the arterial part of the terminal vascular supply has been advocated for quantitative analysis of the vascularisation in human tumours (Mlynek *et al.*, 1985). However, studies in a variety of different tumour types (Monis & Rutenburg, 1960) have shown that sometimes the simultaneous reactivity of stromal connective tissue may preclude the exact evaluation of blood vessels in human cancers.

The capabilities of this histochemical method for the measurement of vascular density were therefore compared with an immunohistochemical technique using a monoclonal antibody directed against endothelium. This study was performed in rectal and oesophageal cancers because in these tumour types pre- and postoperative irradiation is often included in therapeutic regimens.

### Materials and methods

Cryostat sections (5 $\mu$m) of rectal ($n=13$) and oesophageal cancers ($n=17$) and normal mucosa were air-dried, fixed in acetone and stained for alkaline phosphatase (AP) using an azo-dye method. Tissue sections were incubated in a solution containing diazotised triamino-tritolyl-methanechloride (New Fuchsine, CI no. 42520) and naphtol AS-BI phosphate as substrate (Stutte, 1967).

For the immunohistochemical staining of vascular endothelium the monoclonal antibody BW200 was used. This recognises an epitope on an antigen molecule restricted to human neoplastic and non-neoplastic endothelial cells (Alles & Bosslet, 1986). Sections were incubated in turn for 30 min with the BW200 antibody (diluted 1:15; Behring, FRG), peroxidase-conjugated rabbit anti-mouse immunoglobulin (diluted 1:80; Dakopatts, Denmark) and peroxidase-conjugated swine anti-rabbit immunoglobulin (diluted 1:80, Dakopatts, Denmark) at room temperature. Antibodies were diluted in phosphate buffered saline (PBS) containing 20% normal human serum in order to minimise non-specific adsorption. Inhibition of endogeneous peroxidase was achieved by incubating the samples in a glucose-oxidase/glucose mixture (Köller *et al.*, 1986). Between each incubation step sections were extensively washed with PBS. The slides were developed with diaminobenzidine-hydrogen peroxide. Negative controls were performed by substituting PBS or mouse IgG (Sigma, USA) for the primary antibody.

The relative vascular volume ($V$) in sections was quantified by the point-counting method (Chalkley, 1943). In normal tissues, vascular parameters were evaluated in the mucosa and submucosa. It has been shown (Mlynek *et al.*, 1985) that quantification of vascular parameters in normal colorectal mucosa was independent of the spatial orientation of vessels on histological sections. Therefore, only sections cut perpendicularly and not parallel to the luminal surface were used, allowing a simultaneous analysis of vessels in the mucosa and submucosa. Only this spatial orientation of histological sections rendered it possible to analyse the capillary plexus near the basal epithelial cells of normal oesophageal mucosa, which might have been missed on sections cut parallel to the luminal surface. In six patients, comparative microscopic analysis of two randomly oriented histological sections cut from a single tumour block did not reveal a preferential spatial orientation of vessels in tumours.

Sections were examined through a graticule with a regular arrangement of 25 crosses inserted in a 10× eye-piece in combination with a 40× objective. Sections were scanned by systematic sampling (Weibel, 1979). Coincidence of a cross with a vessel was counted as a 'hit'. Forty fields were counted per section. Previous experiments have shown that the evaluation of 40 fields per case sufficed to obtain statistically representative results. In 12 tumours analysis of vascularity was carried out in three different sections taken from peripheral, intermediate and central parts of the tumours.

Assuming that the vessels are cylindrical, vascular surface area ($S=4V\bar{d}/\bar{d}^2$) and vascular length ($L=4V/\pi\bar{d}^2$) per unit volume of tissue were calculated as detailed (Vogel, 1965; Rofstad, 1984). For the calculation of these vascular parameters the diameters ($d$) of 100 vessels cut as closely as possible horizontal to their long axis were collected per case. The occurrence of vessels was recorded and histograms of the distribution in various diameter classes were made. Because not all the vessels demonstrated a pure circular profile, the area equivalent diameter (Weibel, 1979) was recorded with the aid of a semi-automatic analysis system (Zeiss, FRG). Each class vessel diameter was squared, and then the square was multiplied by the frequency of the vessels of that particular diameter. These products were accumulated and divided by the total frequency. The average squared diameter was used to calculate surface area and

Correspondence: R. Porschen, Department of Gastroenterology, University of Düsseldorf, Moorenstr. 5, D-4000 Düsseldorf 1, Federal Republic of Germany.
Received 13 December 1988, and in revised form, 12 April 1989.

**Figure 1** Immunohistochemical staining of the vascular network in normal oesophageal epithelium with the monoclonal antibody BW 200 (**a**) allows an excellent demarcation of vessels (indirect immunoperoxidase technique, counterstained with Mayer's haemalum). The distribution of vessels is comparable to the section stained for alkaline phosphatase (**b**). Original magnification ×160.

length of vessels per $mm^3$ tumour tissue with the formulas given above.

### Results

In normal tissues, the vascular network and especially the distribution of smaller vessels such as capillaries and arterioles could be equally well disclosed by both methods (Figure 1). The monoclonal antibody BW 200 allowed an easy, indubitable identification of capillaries. Endothelial cells demonstrated a strong, homogeneous, linearly arranged immunoreactivity which was pronounced at the cell membrane. This antibody markedly stained endothelia of the arterial side of the vascular network, whereas it reacted more weakly with endothelia of venous vessels. This weaker reactivity did not interfere with the determination of total vascular volume because these vessels were easily identified by their morphology. Staining of parenchymal cells was not observed. The intensity of immunostaining in endothelial cells lining lymphatic capillaries in lymph nodes was much weaker and more granular, contrasting clearly with the homogeneous, linearly arranged staining of blood capillaries.

With the alkaline phosphatase method the endothelium of capillaries and arterioles exhibited a strong red colour product (Figure 1). Endothelial cells of larger veins and arteries sometimes showed a faint reaction product. Occasionally, cells in the basal layer of the oesophageal epithelium also reacted slightly. A patchy colour reaction sometimes occurred in the lamina propria of the rectal mucosa.

The regular vascular architecture seen in normal tissues was abolished in tumours in favour of an irregular, often tortuous pattern which did not follow a preferential spatial orientation. The monoclonal antibody BW 200 (Figure 2) allowed the quantitative immunohistochemical evaluation of vascular parameters in all neoplasms and in normal rectal and oesophageal tissue (Table I). In general, considerable differences in vascularity can be noticed between the individual tumours (Table II). In those tumours in which sections out of different tumour areas were investigated, a marked but statistically insignificant heterogeneity of the vascular supply was noted. Values of vascular density in the mucosa and submucosa of normal tissues (Table I) exceeded those of malignant tissues by a factor of about 1.6. Thus, the normal tissue was richer in vessels than the tumour tissue. The coefficient of variation of vascular parameters in normal tissue was considerably smaller than in malignant tissue. In one poorly differentiated oesophageal cancer, tumour cells exhibited a spotted cytoplasmic reactivity with this antibody directed against an endothelial epitope. This cytoplasmic staining could also be observed on the section developed with the AP method. Using the AP method, sporadic staining of tumour cells could be demonstrated in six patients. Alkaline phosphatase activity was unhomogeneously distributed in the stromal elements of 25 tumours (Figure 2). This activity ranged from a faint to an intense red and intermingled with positive vessels. The positive connective tissue rendered the exact quantification of vascular density impossible in one-third of all the tumours investigated.

**Table I** Capillary volume (CV), total vessel volume (TVV), vascular surface area (S) and length (L) in normal and carcinomatous tissue of the rectum and the oesophagus determined with an immunoperoxidase method using the monoclonal antibody BW 200

| *Tissue* | *CV* (%) | *TVV* (%) | *S* ($mm^2 mm^{-3}$) | *L* ($mm mm^{-3}$) |
|---|---|---|---|---|
| Oesophagus | | | | |
| normal | 3.3±0.7 | 4.5±1.1 | 5.9±2.2 | 105.0±58.0 |
| | (2.1–4.0) | (2.4–5.6) | (3.1–9.7) | (59–208) |
| malignant | 2.0±1.1 | 3.6±1.8 | 3.6±1.6 | 50.4±22.0 |
| | (0.4–4.8) | (1.1–7.2) | (1.5–6.3) | (19–110) |
| Rectum | | | | |
| normal | 3.4±0.8 | 5.9±1.1 | 5.6±1.7 | 70.0±23.5 |
| | (2.4–4.7) | (4.4–7.3) | (3.8–8.4) | (44–101) |
| malignant | 2.1±1.2 | 3.4±1.3 | 3.6±1.6 | 53.3±26.4 |
| | (0.6–5.1) | (1.6–6.0) | (1.7–7.1) | (24–118) |

Mean values (±1 standard deviation) and range are given.

### Discussion

The response of tumours to irradiation depends on the distribution of oxygen which is determined in part by the architecture of the vascular network in tumours. Although a variety of techniques – including histochemical detection of vessel walls and erythrocytes, injection of ink or contrast material and immunohistochemical demonstration of endothelial cells – have been proposed for investigations of vascular density precise data on the vascularisation of human cancers remain sparse. Injection techniques cannot easily be applied to studies of the microcirculation of malignant tumours in human beings. Studies relying on the histochemical staining of erythrocytes suffer from the serious drawback that small vessels can be overlooked because they frequently collapse during the preparation of the tissue.

Alkaline phosphatase in endothelial cells can be demonstrated using an azo-dye method (Stutte, 1967). Alkaline phosphatase activity appears to be intense only in the end-arterial and capillary endothelium while the walls of the other vessels show either little or no enzyme activity, and it

**Figure 2** Vessels in an adenocarcinoma of the rectum react intensely with the monoclonal antibody BW 200 (**a**) and can be easily identified (indirect immunoperoxidase method, counterstained with Mayer's haemalum, original magnification ×200). However, the section stained for alkaline phosphatase demonstrated an intense stromal reaction (**b**), thus precluding the exact identification and quantification of the vascular supply with the histochemical method in this patient.

has been stated that the vascularisation in normal and malignant tissues of the rectum can be reliably analysed by this histochemical method (Mlynek *et al.*, 1985). In our investigation, the regular pattern of the microcirculation in normal tissues was easily identifiable. However, parts of the newly formed connective tissue in tumours also exhibited strong enzyme activity, thus impairing the exact quantification of vascular parameters in a high percentage of tumours. Our results are in accordance with a study investigating a number of different tumour types (Monis & Rutenburg, 1960). In malignant tumours of epithelial origin, stromal activity of alkaline phosphatase was a constant finding. The presence of similar or greater stromal activity in benign tumours and inflammatory processes suggested that the activity of alkaline phosphatase is not related to invasive properties of neoplasms but reflects the higher enzyme content in young stromal cells compared to adult or resting cells.

Our comparative analysis has shown that the monoclonal antibody BW 200 facilitates the recognition of capillaries and arterioles, thus enabling the exact quantification of vascular parameters in normal and malignant tissues. This antibody was produced by immunisation of Balb/c mice with the oat-75 small-cell lung carcinoma cell line and immunoprecipitates a 200 kDa band (Alles & Bosslet, 1986). The observation of a spotted cytoplasmic reactivity in the epithelial cells of one oesophageal cancer with both methods may reflect a close relationship of this carcinoma to vascular differentiation or the existence of a tumour epitope recognised by the antibody BW 200. However, this cytoplasmic reactivity did not impair the evaluation of vascular density in this tumour. Despite its similar molecular weight to factor VIII-related antigen the antigen detected by the antibody BW 200 was shown to be different. In our experience (unpublished personal observation) capillaries demonstrated a stronger immunoreactivity with the monoclonal antibody BW 200 than with an antibody to factor VIII-related antigen (Behring, FRG). In contrast to the monoclonal antibody PAL-E (Schlingemann *et al.*, 1985) the monoclonal antibody BW 200 can also be applied to formaldehyde-fixed and paraffin-embedded tissues, thus allowing retrospective analysis of vascularisation in correlation to clinical outcome in patients with malignant diseases.

The lectin Ulex europaeus I agglutinin has also been suggested for investigation of vascularisation, because it binds to vascular endothelium in malignant and benign human tissues. However, this lectin is not a specific marker for the selective analysis of blood vessels, because staining of lymphatic vessels (Fujme *et al.*, 1984), squamous epithelia (Holthöfer *et al.*, 1982) and colorectal carcinomas (Matsushita *et al.*, 1985) has been described.

Our values reported here for vascular volume, surface area and length are in accordance with those reported for large transplantable adenocarcinomas (Vogel, 1965) and human melanoma xenografts (Rofstad, 1984). Comparable differences in vascularity between normal and malignant tissues were noted by Mlynek *et al.* (1985).

The histopathological analysis of tumours presented here yields information concerning the magnitude of the vascular density. However, the supply of oxygen and nutrients to tumours probably does not only depend on the density of vessels, but is surely influenced by functional parameters, e.g. blood flow and oxygen saturation, and it is obvious that information obtained by morphological studies of the vascular network does not automatically reflect the functional status of the vessels. However, studies of xenografts grown in nude mice demonstrated that the extent of vascularisation derived from morphological analysis paralleled the growth rate of tumours (Rofstad, 1984) and the volume fraction of necrosis (Solesvik *et al.*, 1982). In addition, retrospective analyisis of cervical and nasopharyngeal carcinomas yielded a statistically significant correlation between vascular density and survival time after radiotherapy (Siracká *et al.*, 1988; Delides *et al.*, 1988). Therefore, it can be concluded that morphometric analysis of vascularity can serve as an important indicator of the functional status of the vascular supply and of oxygenation of neoplastic tissue.

**Table II** Percentage of capillary volume (CV) and of total vessel volume (TVV) evaluated by point counting on histological sections of 13 rectal and 17 oesophageal carcinomas stained with the monoclonal antibody BW 200

| *Oesophageal carcinoma* | | | *Rectal carcinoma* | | |
|---|---|---|---|---|---|
| *Case no.* | *CV (%)* | *TVV (%)* | *Case no.* | *CV (%)* | *TVV (%)* |
| 1 | 0.9 | 1.4 | 18 | 1.6 | 1.9 |
| 2 | 2.0 | 2.5 | 19 | 5.1 | 6.0 |
| 3 | 1.9 | 5.0 | 20 | 1.3 | 2.9 |
| 4 | 1.9 | 3.9 | 21 | 3.2 | 4.8 |
| 5 | 1.1 | 3.1 | 22 | 2.3 | 2.8 |
| 6 | 1.7 | 2.4 | 23 | 1.9 | 4.7 |
| 7 | 1.6 | 2.0 | 24 | 3.4 | 4.0 |
| 8 | 4.0 | 7.2 | 25 | 2.6 | 4.5 |
| 9 | 2.0 | 4.9 | 26 | 1.2 | 2.9 |
| 10 | 1.4 | 1.9 | 27 | 1.2 | 1.8 |
| 11 | 0.4 | 2.5 | 28 | 0.9 | 3.7 |
| 12 | 1.1 | 1.1 | 29 | 2.5 | 3.2 |
| 13 | 1.7 | 2.3 | 30 | 0.6 | 1.6 |
| 14 | 2.1 | 5.5 | | | |
| 15 | 2.2 | 4.3 | | | |
| 16 | 2.4 | 4.8 | | | |
| 17 | 4.8 | 6.6 | | | |

In conclusion, application of the monoclonal antibody BW 200 facilitates the identification of vessels – especially capillaries – in neoplastic tissues and is superior to histochemical studies using the alkaline phosphatase method. It provides a precise technique for the calculation of vascular density and investigations of the importance of tumour blood supply in man.

The excellent technical assistance of Mrs V. Stönner is gratefully acknowledged. This study was supported by a grant from the Dr Mildred Scheel Foundation for Cancer Research.

### References

ALLES, J.U. & BOSSLET, K. (1986). Immunohistochemical and immunochemical characterization of a new endothelial cell – specific antigen. *J. Histochem. Cytochem.*, **34**, 209.

CHALKLEY, H.W. (1943). Method for the quantitative morphologic analysis of tissues. *J. Natl Cancer Inst.*, **4**, 47.

DELIDES, G.S., VENIZELOS, J. & RÉVÉSZ, L. (1988). Vascularization and curability of stage III and IV nasopharyngeal tumors. *J. Cancer Res. Clin. Oncol.*, **114**, 321.

FUJIME, M., LIN, C.W. & PROUT, G.R. (1984). Identification of vessels by lectin-immunoperoxidase staining of endothelium: possible applications in urogenital malignancies. *J. Urol.*, **131**, 566.

HOLTHÖFER, H., VIRTANEN, I., KARINIEMI, A.L., HORMIA, M., LINDER, E. & MIETINNEN, A. (1982). Ulex europaeus I lectin as a marker for vascular endothelium in human tissues. *Lab. Invest.*, **47**, 60.

KÖLLER, U., STOCKINGER, H., MAJDIC, O., BETTELHEIM, P. & KNAPP, W. (1986). A rapid and simple immunoperoxidase staining procedure for blood and bone marrow samples. *J. Immunol. Methods*, **86**, 75.

MATSUSHITA, Y., YONEZAWA, S., NAKAMURA, T. and 4 others (1985). Carcinoma-specific Ulex europaeus agglutinin I binding glycoproteins of human colorectal carcinoma and its relation to carcinoembryonic antigen. *J. Natl Cancer Inst.*, **75**, 219.

MLYNEK, M.L., VAN BEUNINGEN, D., LEDER, L.D. & STREFFER, C. (1985). Measurement of the grade of vascularisation in histological tumour tissue sections. *Br. J. Cancer*, **52**, 945.

MONIS, B. & RUTENBURG, A.M. (1960). Alkaline phosphatase activity in neoplastic and inflammatory tissues of man. *Cancer*, **13**, 538.

ROFSTAD, E.K. (1984). Growth and vascular structure of human melanoma xenografts. *Cell Tissue Kinet.*, **17**, 91.

SCHLINGEMANN, R.O., DINGJAN, G.M., EMEIS, J.J., BLOK, J., WARNAAR, S.O. & RUITER, D.J. (1985). Monoclonal antibody PAL-E specific for endothelium. *Lab. Invest.*, **52**, 71.

SIRACKÁ, E., RÉVÉSZ, L., KOVÁC, R. & SIRACKY, J. (1988). Vascular density in carcinoma of the uterine cervix and its predictive value for radiotherapy. *Int. J. Cancer*, **41**, 819.

SOLESVIK, O.V., ROFSTAD, E.K. & BRUSTAD, T. (1982). Vascular structure of five human malignant melanomas grown in athymic nude mice. *Br. J. Cancer*, **46**, 557.

STUTTE, H.J. (1967). Hexazotiertes Triamino-Tritolyl-Methanchlorid (Neufuchsin) als Kupplungssalz in der Fermenthistochemie. *Histochemie*, **8**, 327.

VOGEL, A.W. (1965). Intratumoral vascular changes with increased size of a mammary adenocarcinoma: new methods and results. *J. Natl Cancer Inst.*, **34**, 571.

WEIBEL, E.R. (1979). *Stereological Methods, Vol. 1.* Academic Press: London.